# Change of Deformation Mechanisms Leading to High Strength and Large Ductility in Mg-Zn-Zr-Ca Alloy with Fully Recrystallized Ultrafine Grained Microstructures

**DOI:** 10.1038/s41598-019-48271-5

**Published:** 2019-08-12

**Authors:** Ruixiao Zheng, Tilak Bhattacharjee, Si Gao, Wu Gong, Akinobu Shibata, Taisuke Sasaki, Kazuhiro Hono, Nobuhiro Tsuji

**Affiliations:** 1 School of Materials Science and Engineering, Beihang University, Beijing, 100191 China; 2 Department of Materials Science and Engineering, Kyoto University, Yoshida Honmachi, Sakyo-ku, Kyoto 606-8501 Japan; 3 Elements Strategy Initiative for Structural Materials (ESISM), Kyoto University, Yoshida Honmachi, Sakyo-ku, Kyoto 606-8501 Japan; 4 National Institute for Materials Science, 1-2-1 Sengen, Tsukuba, 305-0047 Japan

**Keywords:** Metals and alloys, Mechanical properties

## Abstract

Recently, we have found that fully recrystallized ultrafine-grained (UFG) microstructures could be realized in a commercial precipitation-hardened Magnesium (Mg) alloy. The UFG specimens exhibited high strength and large ductility under tensile test, but underlying mechanisms for good mechanical properties remained unclear. In this study, we have carried out systematic observations of deformation microstructures for revealing the influence of grain size on the change of dominant deformation modes. We found that plastic deformation of conventionally coarse-grained specimen was predominated by {0001} <11–20> slip and {10–12} <10–11> twinning, and the quick decrease of work-hardening rate was mainly due to the early saturation of deformation twins. For the UFG specimens, {10–12} <10–11> twinning was dramatically suppressed, while non-basal slip systems containing <*c*> component of Burgers vector were activated, which contributed significantly to the enhanced work-hardening rate leading to high strength and large ductility. It was clarified by this study that limited ductility of hexagonal Mg alloys could be overcome by activating unusual slip systems (<*c* + *a*> dislocations) in fully recrystallized UFG microstructures.

## Introduction

As the lightest metal, magnesium (Mg) and its alloys have long been considered as candidate materials to be used in aerospace and automobile industries^1–5^. However, so far, the applications of Mg and Mg alloys are substantially restricted and most of them are used in cast products. One of the reasons to hinder their widespread applications is the low strength, which is much lower than that of other metallic materials widely used. Another critical shortcoming is their poor ductility and formability. Unlike other metallic materials, such as steels and Al alloys having cubic crystal structures, Mg has a hexagonal close-packed (HCP) crystal structure with an axial ratio (***c***/***a***) of 1.624. As a result, only {0001} <11–20> basal slip system having <***a***> Burgers vector is easily activated at room temperature. However, basal slip can realize only 2-dimentional deformation in each grain, which is far less than the 5 independent slip systems required for free deformation in polycrystalline materials in von Mises criterion, resulting in limited cold-forming capability as well as pronounced mechanical anisotropy in Mg alloys^6–8^. Deformation twinning is another important deformation mechanism in addition to basal slip, which can accommodate strains along the ***c***-axis during plastic deformation^9,10^. However, the deformation twins usually induce the formation of cracks or voids at grain boundaries they impinge, leading to failure^11^. Therefore, a lot of efforts have been paid to improve the strength and ductility/formability in Mg alloys.

Processing at elevated temperatures is one possible solution for improving the formability of Mg alloys, since multiple slip systems in addition to basal slip can be activated at elevated temperatures because of the temperature dependence of critical resolved shear stress (CRSS) for each slip system^12^. However, high temperature processes cost and may lead to grain coarsening. Furthermore, intermediate annealing is often required during multi-pass high temperature deformation to avoid cracks, which increases the processing cost furthermore.

On the other hand, Mg alloys can be deformed to ultra-high strains without cracking by severe plastic deformation (SPD) at relatively low or even ambient temperature^13–15^. For instance, Mukai *et al*.^15^ found that the grain size of a commercial AZ31 Mg alloy could be refined to 1 μm after heavy deformation by equal channel angular pressing (ECAP) for 8 passes (effective strain ~9.2) at a temperature of 200 °C. Moreover, Mukai *et al*.^15,16^ have also demonstrated that the ductility in AZ31 and WE43 Mg alloys was significantly enhanced by the grain refinement. Recently, we have reported that the average grain size of a precipitation hardened Mg alloy (ZKX600) could be refined to 100 nm after SPD using high pressure torsion (HPT) by 1 rotation (360°) at room temperature. Furthermore, fully recrystallized ultrafine grained (UFG) structures which have not been achieved by other researchers were also obtained in the HPT processed material after subsequent rapid annealing^17^.

It has been known that plastic instability happens in most UFG materials at early stages of tensile deformation because of highly enhanced yield strength and limitation of strain hardening in UFG structures, and thus it is difficult to manage both high strength and enough ductility in bulk UFG materials. For example, UFG Al and IF steel having sub-micrometer grain sizes exhibited quite early plastic instability, so that their uniform elongation is limited within a few percents^18^. In contrast, fully-recrystallized UFG ZKX600 alloy specimens exhibited both enhanced strength and ductility compared to those of the starting material having conventional coarse grained microstructure^17^. However, detailed strengthening and toughening mechanisms have not been clarified yet. Thus, the main purpose of this paper is to reveal the influence of grain size on the change of deformation mechanism in the ZKX600 alloy. We carefully observed the deformed microstructures of the specimens having two typical grain sizes and found that the dominant deformation mechanisms (*i.e*., activity of slip systems and deformation twinning) have been significantly changed in the UFG specimen.

## Results

### Microstructures and tensile properties

**Figure 1:**
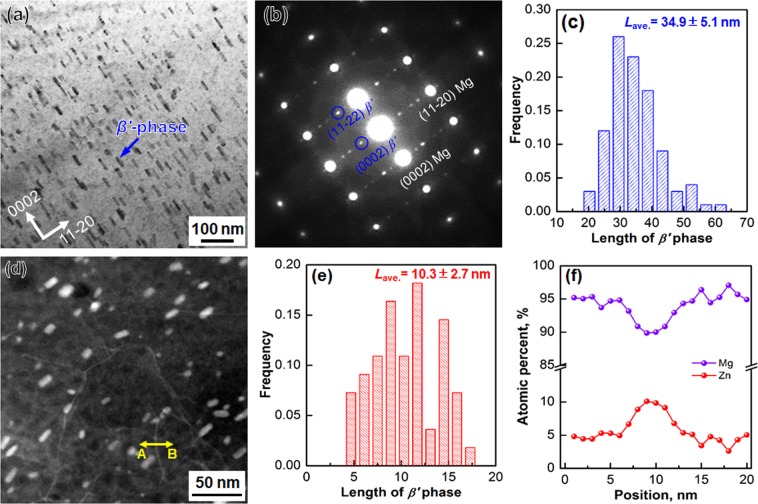
Microstructure of (**a**–**c**) the as-ST and (**d**–**f**) the 360° (γ = 23.55) HPT processed ZKX600 specimens. (**a**) BF-TEM image showing high density of nano-precipitates, and (**b**) corresponding selected area electron diffraction (SAED) pattern of (**a**). (**c**) Histogram showing the length distribution of nano-precipitates observed in (**a**) statistically. (**d**) HAADF-STEM image of the 360° HPT processed specimen showing refined nano-precipitates and grain boundary segregation of Zn. (**e**) Statistical histogram showing the length distribution of the nano-precipitates observed in (**d**). (f) EDS line profiles of Mg and Zn atoms carried out along a line across a grain boundary marked by the yellow arrow A-B in (**d**).

As mentioned in the last section, the ZKX600 alloy is a typical precipitation hardened Mg alloy. The change of nano-precipitates during the present processing was investigated by transmission electron microscopy (TEM). Figure 1(a) shows a bright field (BF)-TEM image of the as-solution treated (as-ST) sample observed along [10-10] zone axis. High density of rod-shaped nano-precipitates retained. Selected area electron diffraction (SAED) pattern (Fig. 1(b)) suggested that the majority of the precipitates were β’_1_ phase, whose structure was a hexagonal structure as reported previously in various Mg-Zn based alloys^19^. Statistical length distribution of the rod-shaped precipitates is shown in Fig. 1(c), and their average length was 34.9 ± 5.1 nm.

Microstructure of the 360° HPT processed specimen was observed by TEM in high angle annular dark field (HAADF) scanning transmission electron microscopy (STEM) mode (Fig. 1(d)). Since the heavier atoms show brighter contrast than the lighter ones in the HAADF-STEM mode, two kinds of microstructural features can be identified. Firstly, the size of β′_1_ phase was refined by HPT. The histogram of the length of the precipitates (Fig. 1(e)) indicated that the average length of β′_1_ phase in the 360° HPT processed specimen was 10.3 ± 2.7 nm, which was about one-third of that in the as-ST specimen. As the second feature, the HAADF-STEM image (Fig. 1(d)) showed bright contrast at grain boundaries, suggesting segregation of alloying elements occurred dynamically during HPT. Figure 1(f) shows EDS line profiles of Mg and Zn atoms along a line (marked by yellow arrow A-B) across a grain boundary in the HAADF-STEM image (Fig. 1(d)). The concentration of Zn at the grain boundary was about 10 at.%, which was almost twice as that in the matrix (~5 at.%), demonstrating a clear segregation of Zn at deformation induced grain boundaries. Similar phenomena have been also reported in a HPT processed Mg-Zn-Y alloy by Basha *et al*.^20^ previously.

**Figure 2:**
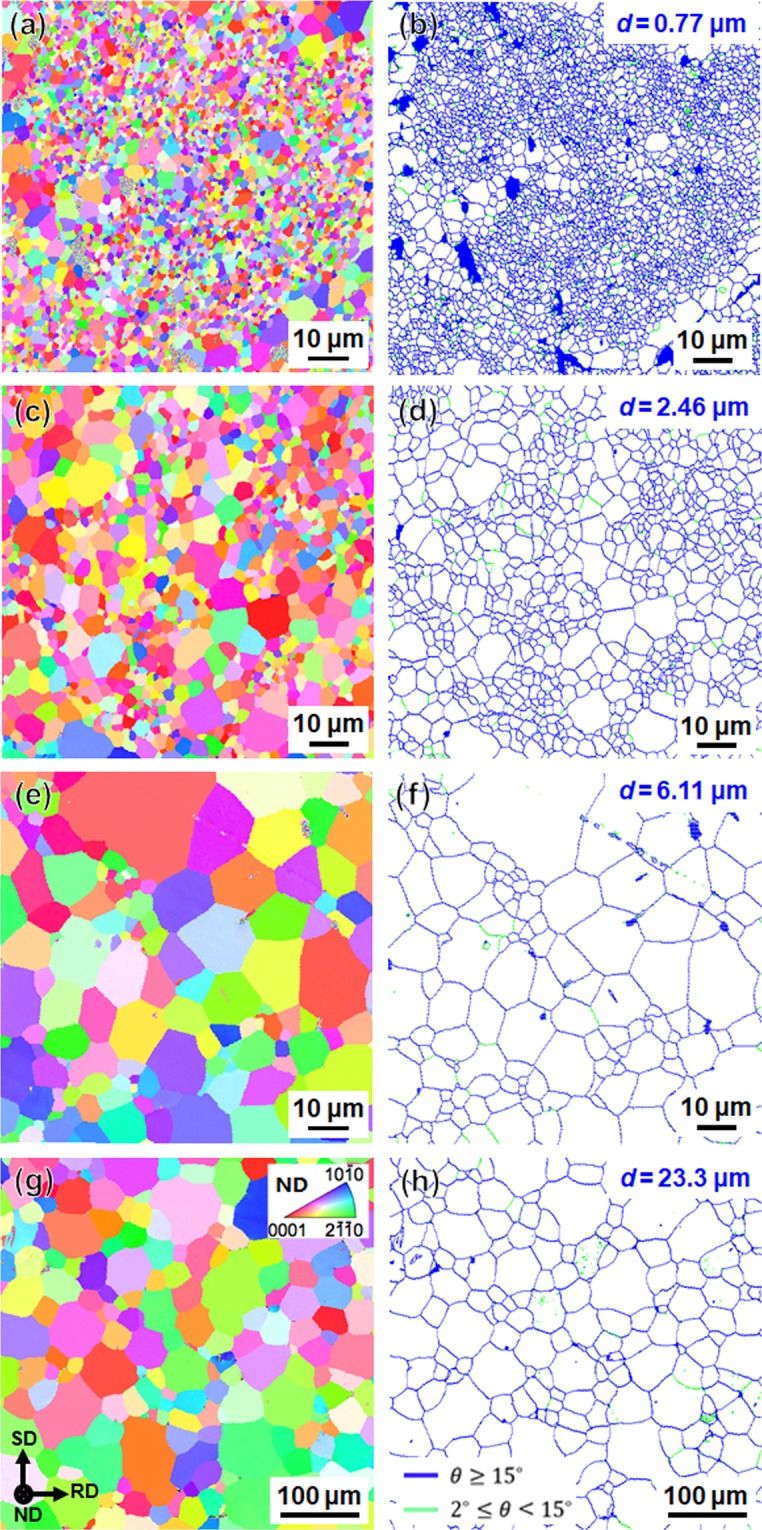
EBSD-IPF maps (**a**,**c**,**e**,**g**) and corresponding GB maps (**b**,**d**,**f**,**h**) of the specimens HPT processed by 360° (γ = 23.55) and then annealed at various temperatures for different periods. (**a**,**b**) Annealed at 300 °C for 1 min. (**c**,**d**) Annealed at 400 °C for 30 min. (**e**,**f**) Annealed at 450 °C for 30 min. (**g**,**h**) Annealed at 500 °C for 30 min. The colors in the IPF maps indicate crystallographic orientations parallel to the normal direction of the HPT discs. The blue and green lines in the GB maps correspond to high angle grain boundaries (HAGBs) with misorientation angles larger than 15° and low angle grain boundaries (LAGBs) with misorientation angles between 2° and 15°, respectively.

It has been clarified by our previous study that saturated UFG microstructure could be realized after HPT by 360° rotation^17^. Thus, in the present study, the specimen HPT processed by 360° (corresponding shear strain: γ = 23.55) was annealed at various temperatures for different holding periods, and then the specimens with recrystallized grain sizes ranging from sub-micrometer to several tens of micrometers were obtained. The EBSD inverse pole figure (IPF) maps and corresponding grain boundary (GB) maps of several representative specimens are displayed in Fig. 2. The specimen with the smallest recrystallized grain size of 0.77 μm was obtained by rapid annealing for 1 minute at 300 °C, as shown in Fig. 2(a),(b). With increasing the annealing temperature and time, grain size was coarsened. The largest grain size obtained in this study was 23.3 μm in the specimen annealed for 30 minutes at 500 °C.

Texture evolution in the specimens shown in Fig. 2 was also investigated. Lee *et al*.^21^ have studied the texture evolution in a ZK60A Mg alloy during HPT and they found that a significant difference in the evolution of texture between the overall texture taken throughout the disc and the local texture at the disk edges up to at least 5 turns of HPT. That means, the selection of the measured area is critical for texture analysis in HPT processed Mg. In the present study, local areas corresponded to the center of the gauge part of the tensile specimens (see Fig. S2(c)) were subjected for the local texture analysis using EBSD, so that the measured areas were around a particular radial position in the HPT discs. Obtained (0002) and (10-10) pole figures are presented in Fig. S1. Note that a sufficient number of grains were included (e.g., more than 5000 grains for the UFG specimens and more than 1000 grains for the specimens having mean grain sizes of several tens of micrometers) to ensure the reliability of the results. As shown in Fig. S1, for all the specimens, the peak intensity regions located far from the center of the (0001) pole figures, indicating that the basal texture usually observed in rolled or extruded Mg alloys even after annealing was not formed in the present HPT processed and annealed specimens. It could be considered that the weak textures in the annealed specimens in the present study was due to the co-addition of Zn and Ca^22,23^. Zeng *et al*.^23^ have investigated the effect of alloying elements on recrystallization texture in a cold rolled and annealed Mg-Zn-Ca ternary alloy. They found that Zn and Ca atoms segregated strongly at high-energy boundaries of the recrystallized grains, and the grain growth in Mg-Zn-Ca was much more suppressed than that in the Mg-Zn and Mg-Ca binary alloys. They concluded that the co-segregation significantly reduced the boundary mobility probably by decreasing grain boundary energy, leading to an uniform growth of recrystallized grains having various orientations.

**Figure 3:**
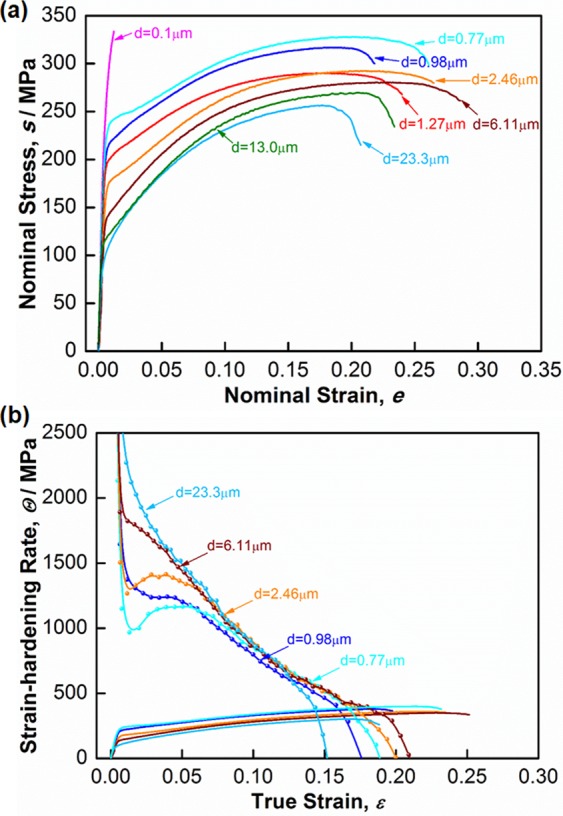
Tensile properties of the specimens with various mean grain sizes. (**a**) Nominal stress-strain curves (reproduce from^25^). (**b**) Curves showing work-hardening rate and true stress against true strain of representative specimens having different grain sizes.

Nominal stress-strain curves of the as-deformed and the as-annealed specimens are displayed in Fig. 3(a). As we have reported previously^24,25^, the yield strength (YS) of the 360° HPT processed specimen (*d* = 0.1 μm) reached 311 ± 3 MPa. However, premature fracture took place at a very limited total elongation (TE) of 1.2 ± 0.1% (in nominal strain). The low ductility was primarily due to their very high YS and low work-hardening capability, which was caused by their very small grain sizes and high density of dislocations retained in the microstructure^18,26^. Interestingly, fully recrystallized UFG specimens could manage both high strength and large ductility. Particularly, the specimen with the finest recrystallized grain size (*d* = 0.77 μm) exhibited superior ductility (uniform elongation (UE)~20.5 ± 1.0%, TE~26.1 ± 1.4%), without sacrificing high ultimate tensile strength (UTS, ~328 ± 3 MPa). With increasing the grain size, the strength decreased gradually, but the change in tensile elongations was not significant. For example, the UE and TE were maintained at 22.7 ± 1.8% and 28.0 ± 1.3%, respectively, in the specimen having a mean grain size of 6.11 μm. Strength and ductility decreased simultaneously by further increase of the grain size. The YS and TE decreased to 90 ± 5 MPa and 20.3 ± 2.0%, respectively, in the specimen having a coarse grain size of 23.3 μm. It is worth pointing out that, in addition to grain growth, coarsening of precipitates (β′_1_ phase) may take place during the long-time annealing treatment at high temperatures, which can also result in the decrease of the tensile strength.

It is well known that the large tensile ductility is often derived from the high work-hardening rate (*Θ* = d*σ*/d*ε*, where *σ* and *ε* are the true stress and true strain, respectively), since uniform elongation in tensile tests is determined by the plastic instability condition (such as, Considère criterion: *Θ* ≦ *σ*). The *Θ*-*ε* curves and *σ*-*ε* curves of several representative specimens are plotted in Fig. 3(b). The intersection points between those curves correspond to the point of plastic instability. As shown in Fig. 3(b), the UFG specimens showed discontinuous work-hardening behaviors at small strains. The *Θ* decreased quickly at very beginning of plastic deformation, followed by an increase of *Θ* upon further deformation, resulting in a V-shaped work-hardening curve at small strains (below 0.03). After that, the *Θ* value became higher than that of the coarse grained specimen (*d* = 23.3 μm), leading to relatively larger uniform elongation. As the grain size increased, the V-shaped valley became less and less obvious and eventually disappeared.

**Figure 4:**
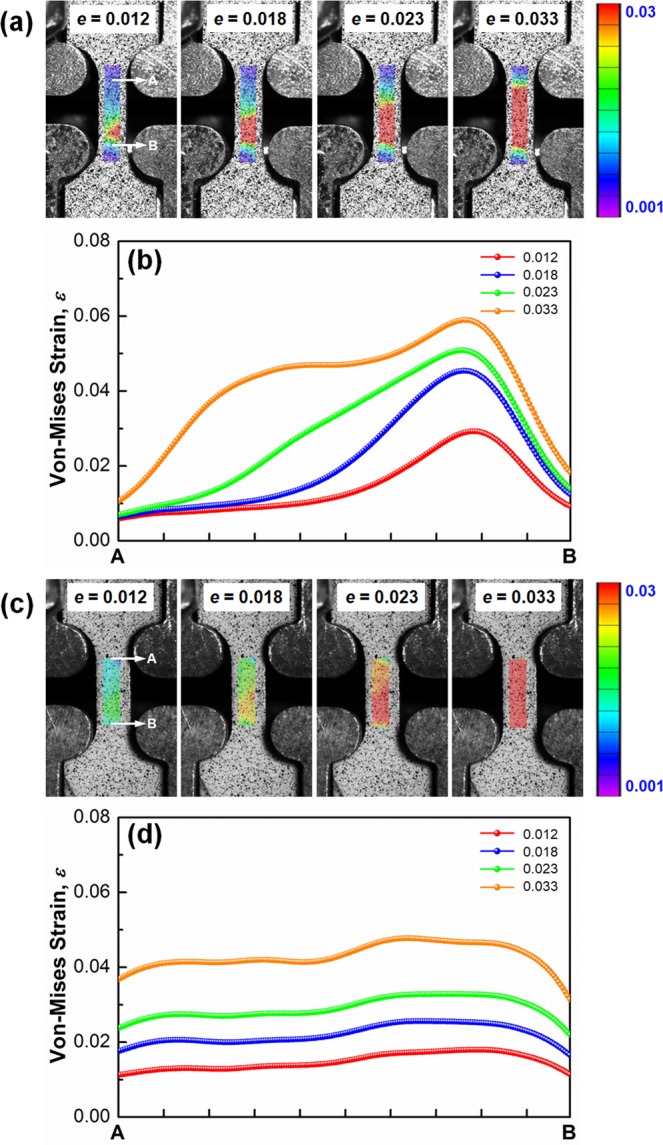
Distributions of local von-Mises strains within the gage part of the tensile specimens having mean grain sizes of 0.77 μm (**a**,**b**) and 23.3 μm (**c**,**d**), respectively. The line profiles (**b**,**d**) showing quantitative strain distributions along the gage length measured from the DIC local strain maps shown in (**a**,**c**), respectively.

It is considered that the V-shaped change of the work-hardening rate at small strains in the UFG specimens was related to the discontinuous yielding phenomena involving Lüders deformation^27,28^. The local von-Mises strain distributions of a UFG specimen (*d* = 0.77 μm) and a coarse grained specimen (*d* = 23.3 μm) at various tensile strains were analyzed by a digital image correlation (DIC) technique^29^. As shown in Fig. 4(a), a clear strain localization can be observed at a lower region of the gage in the UFG specimen at a tensile strain of 0.012. The strain localized band started to propagate to upper side upon further deformation, and the local strain distribution within the gage part became uniform at a strain of 0.033. The line profiles shown in Fig. 4(b) show quantitative distributions of local strains along the gage length measured from the DIC local strain maps (Fig. 4(a)), which confirms the appearance and propagation of Lüders band (strain-localized band). In contrast, the strain distribution within the gage part in the coarse grained specimen was rather homogeneous at all strains (Fig. 4(c),(d)), which corresponded to the continuous yielding on the stress-strain curve (Fig. 4(a)) as well as monotonic decrease in the work-hardening rate curve (Fig. 4(b)). From these results, it could be concluded that the V-shaped work-hardening curves in the UFG specimens (Fig. 4(b)) were attributed to the discontinuous yielding characterized by the yield-drop and following initiation and propagation of Lüders band in those specimens.

### Deformation mechanisms of coarse grained (CG) specimen

**Figure 5:**
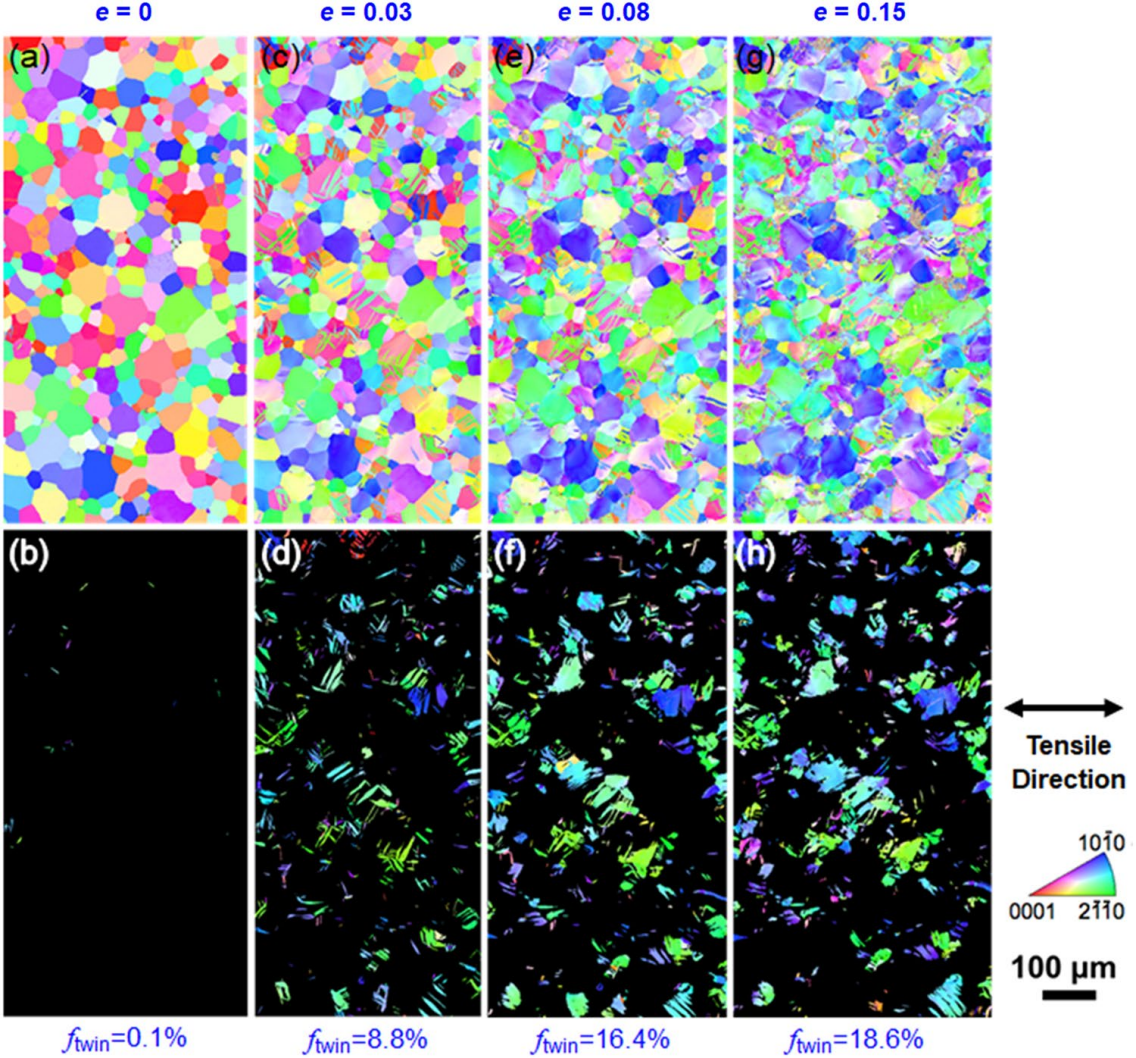
Identical area observation by EBSD of the coarse grained specimen (*d* = 23.3 μm) after tensile deformation to specified strains. Upper row (**a**,**c**,**e**,**g**): EBSD-IPF maps of the whole area at various strains. Lower row (**b**,**d**,**f**,**h**): EBSD-IPF maps extracting only the twinned areas. The colors in the IPF maps indicate crystallographic orientation parallel to the tensile direction (horizontal direction of the figures).

A CG specimen (*d* = 23.3 μm) were selected for detailed investigations on deformed microstructures. First, we focused on deformation twinning. Figure 5 shows EBSD-IPF maps of an identical area in the CG specimen at different tensile strains (0, 0.03, 0.08 and 0.15). The upper images are the full IPF maps showing overall microstructures, and the lower images are the IPF maps extracting only deformation twins. The orientation distribution of the initial microstructure (*e* = 0) was rather random. Besides, only a few {10–12} <10–11> twins (*f*_twin_ = 0.1%) were observed, which were considered to be introduced by mechanical polishing. At a tensile strain of 0.03, a fairly large number of lenticular-shaped deformation twins (*f*_twin_ = 8.8%) were formed. They were all {10–12} <10–11> twins, whose CRSS is significantly lower than other twinning systems in Mg alloys^30^. Since the {10–12} <10–11> twin rotates the lattice by 86°, it can effectively accommodate the extension strain along ***c***-axis^31,32^. In addition, most of the twins appeared in blue or green colors in the partitioned EBSD-IPF maps (Fig. 5(d,f,h)), indicating that the ***c***-axis of the twins was distributed in the direction perpendicular to the tensile direction. In other words, the grains with their ***c***-axis parallel to the tensile direction tended to show {10–12} <10–11> twins. The change of the colors in the upper images, where grains having red color (*i.e*., [0001]//tensile direction) decreased with increasing tensile strain, supported such a tendency. Further deformation to a strain of 0.08, the area fraction of {10–12} <10–11> twins dramatically increased to 16.4%, and mutual intersection of twins were frequently observed. It is interesting that some grains were completely replaced by twins. However, at a strain of 0.15, which was almost twice as that of the previous stage (0.08), there was only a slight increase of the twined area (18.6%), indicating a saturation of deformation twinning around this strain level.

**Figure 6:**
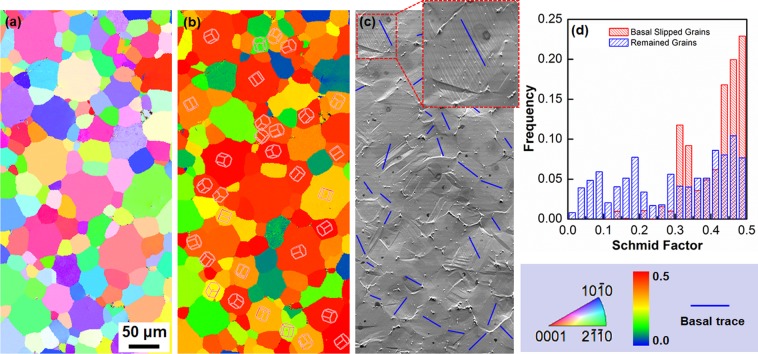
EBSD-assisted slip trace analysis of the coarse grained specimen (*d* = 23.3 μm). (**a**) EBSD-IPF map and (**b**) corresponding Schmid factor (Sc) map for basal slip. The colors in the IPF maps indicate crystallographic orientations parallel to the tensile direction (horizontal direction of the figures). (**c**) SEM image taken in the identical area as (**a**,**b**) after tensile deformation to a tensile strain of 0.03. The inset in (**c**) is an enlarged view of a typical grain showing basal slip lines. (**d**) Statistical histogram showing distributions of Schmid factor of the grains with and without basal slip traces.

Slip activity in the CG specimen at relatively small tensile strains was studied by slip trace analysis. First, a selected area in the specimen was observed by EBSD before the tensile test. An EBSD-IPF map and corresponding Schmid factor map for basal slip are shown in Fig. 6(a),(b), respectively. After a tensile strain of 0.03, the appearance of slip traces in the identical area was tracked by SEM. As shown in Fig. 6(c), sets of parallel slip lines (partly highlighted by blue lines) were clearly observed in many grains. In the Schmid factor map (Fig. 6(b)), projections of hexagonal unit cell corresponding to orientations of grains are superimposed in the grains where clear slip lines were observed. EBSD-assisted slip trace analysis confirmed that all the slip traces appeared in the SEM image were parallel to (0001) traces, indicating they were basal slips. Figure 6(d) shows distributions of Schmid factors of the grains where basal slip lines were observed (red bars) or the grains without basal slip lines (blue bars). Statistical results (Fig. 6(d)) indicated that most of the basal slip traces observed in the grains had Schmid factors larger than 0.3.

**Figure 7:**
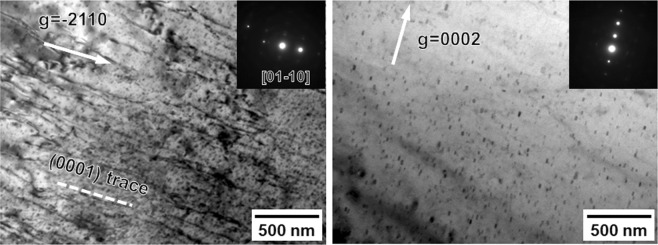
Dislocation structures of a typical grain in the CG specimen (*d* = 23.3 μm) tensile deformed to a strain of 0.10. (**a**) BF-TEM image observed from near the [01–10] zone axis under two beam condition with ***g*** = −2110. (**b**) BF-TEM image observed from near the [01–10] zone axis under two beam condition with ***g*** = 0002.

The surface of the tensile specimen became very rough at higher strain levels and it was difficult to determine the activated slip systems through the EBSD assisted slip trace analysis. Instead, we carried out TEM observations of the CG specimen deformed to a tensile strain of 0.10. The “***g***·***b*** **=** 0” invisibility criterion (where ***g*** is operation vector, ***b*** is Burgers vector) was used to distinguish the types of dislocations (<***a***> type with ***b*** **=** 1/3 <11–20>, <***c***> -including type like <***c*** + ***a***> with ***b*** **=** 1/3 <11–23>)^33,34^. Figure 7(a,b) represent BF-TEM images of an identical grain observed from the near [01–10] zone axis satisfying two beam condition. The operation vector (***g***) in Fig. 7(a,b) were −2110 and 0002, respectively. According to the “***g***·***b*** **=** 0” criterion, dislocations having <***c***> component are invisible in Fig. 7(a), while <***a***> type dislocations should disappear in Fig. 7(b). As shown in Fig. 7(a), profuse dislocations aligned parallel to the basal trace (the broken line in **(a)**) were observed. These dislocations were most likely basal dislocations having <***a***> component in Burgers vector. On the other hand, Fig. 7(b) was totally free of any dislocation contrast, suggesting that non-basal dislocations having <***c***> component in Burgers vector were still not activated. It should be noted that the activation of non-basal dislocations have been reported in some Ca-containing Mg alloys (such as Mg-1Al-0.1Ca alloy reported by Sandlöbes *et al*.)^35,36^. However, those alloys were mostly simple solid solution Mg alloys. In contrast, the distribution of Ca in precipitation hardened Mg alloys were inhomogeneous. For example, in Mg-Zn based alloys, Ca precipitates out with Zn, forming nano-precipitates^37^. Thus, the role of Ca in the present quaternary alloy is somehow complicated and further investigations on this issue should be done in the future.

Therefore, it is considered that the plastic deformation of the CG specimen was predominated by the {10–12} <10–11> twinning and {0001} <11–20> basal slip. The quick decrease of work-hardening rate was mainly due to the rapid growth and saturation of deformation twins, as indicated by the deformation microstructures observed (Figs 5–7**)**.

### Change of deformation mechanisms in fully recrystallized UFG specimen

**Figure 8:**
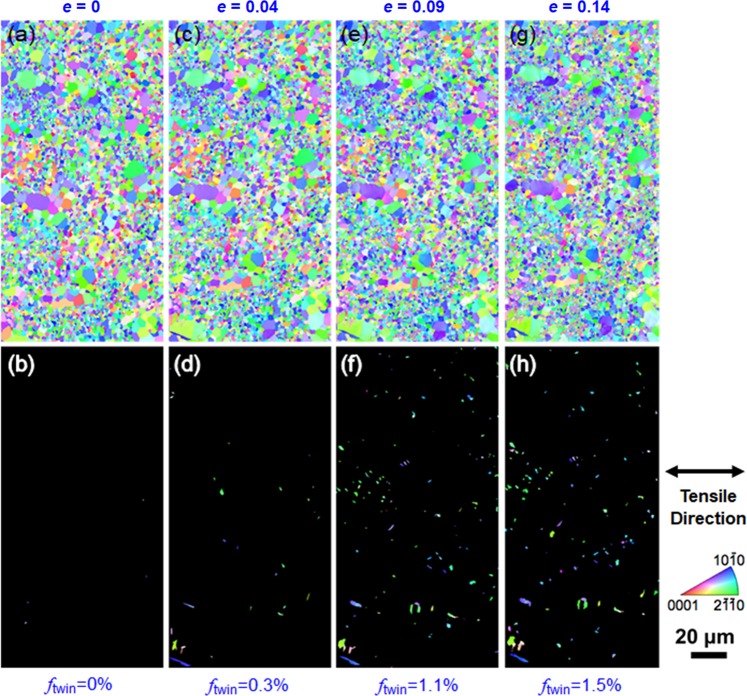
Identical area observation by EBSD of the UFG specimen (*d* = 0.98 μm) after tensile deformation to specified strains. Upper row (**a**,**c**,**e**,**g**): EBSD-IPF maps of the whole area at different strains. Lower row (**b**,**d**,**f**,**h**): EBSD-IPF maps extracting only twinned areas. The colors in the IPF maps indicate crystallographic orientation parallel to the tensile direction (horizontal direction in the figures).

Identical area EBSD observations of deformation twinning was also conducted on the UFG specimen (*d* = 0.98 μm), in the same way as those for the CG specimen (Fig. 5). As shown in Fig. 8, the initial microstructure (*e* = 0) was totally free of twins, suggesting that the grain-refinement strengthening inhibited deformation twinning even during mechanical polishing. At a tensile strain of 0.04, only 0.3% of deformation twins were formed, which was significantly lower than that of the CG specimen (8.8%) at a similar tensile strain (*e* = 0.03). Again, these twins were exclusively {10–12} <10–11> twins. With the increase of tensile strain, the area fraction of twins increased gradually. Even at a strain of 0.14, however, the fraction of twins was only 1.5%, which was again far less than that in the CG specimen (18.6%) deformed to a similar strain.

Figure 8 clearly showed that deformation twinning was dramatically suppressed in the UFG specimen, however the specimen exhibited better ductility than the CG counterpart. If only basal slips were activated, such a good plasticity cannot be realized in the polycrystalline specimen with the ultrafine grain size. Therefore, some unusual deformation mechanisms, such as non-basal slip systems, might be activated in the UFG specimens. For example, Koike *et al*.^38^ reported that a fine grained AZ31 Mg alloy (*d* = 6.5 μm) exhibited very large room temperature tensile elongation of 0.47, which was mainly due to the cross-slip to non-basal planes induced by plastic compatibility. In contrast, Cepeda *et al*.^39^ reported reduced tensile ductility in the pure Mg when grain size was refined from 36 μm to 5 μm. Their slip trace analysis suggested a transition from the non-basal slips dominated deformation in the coarse grained (36 μm) specimen to the basal slip dominated deformation in the fine grained (5 μm) specimen, which was totally opposite to the result reported by Koike *et al*.^38^. Thus, we focused on investigating the active slip systems in the UFG specimen (*d* = 0.98 μm).

**Figure 9:**
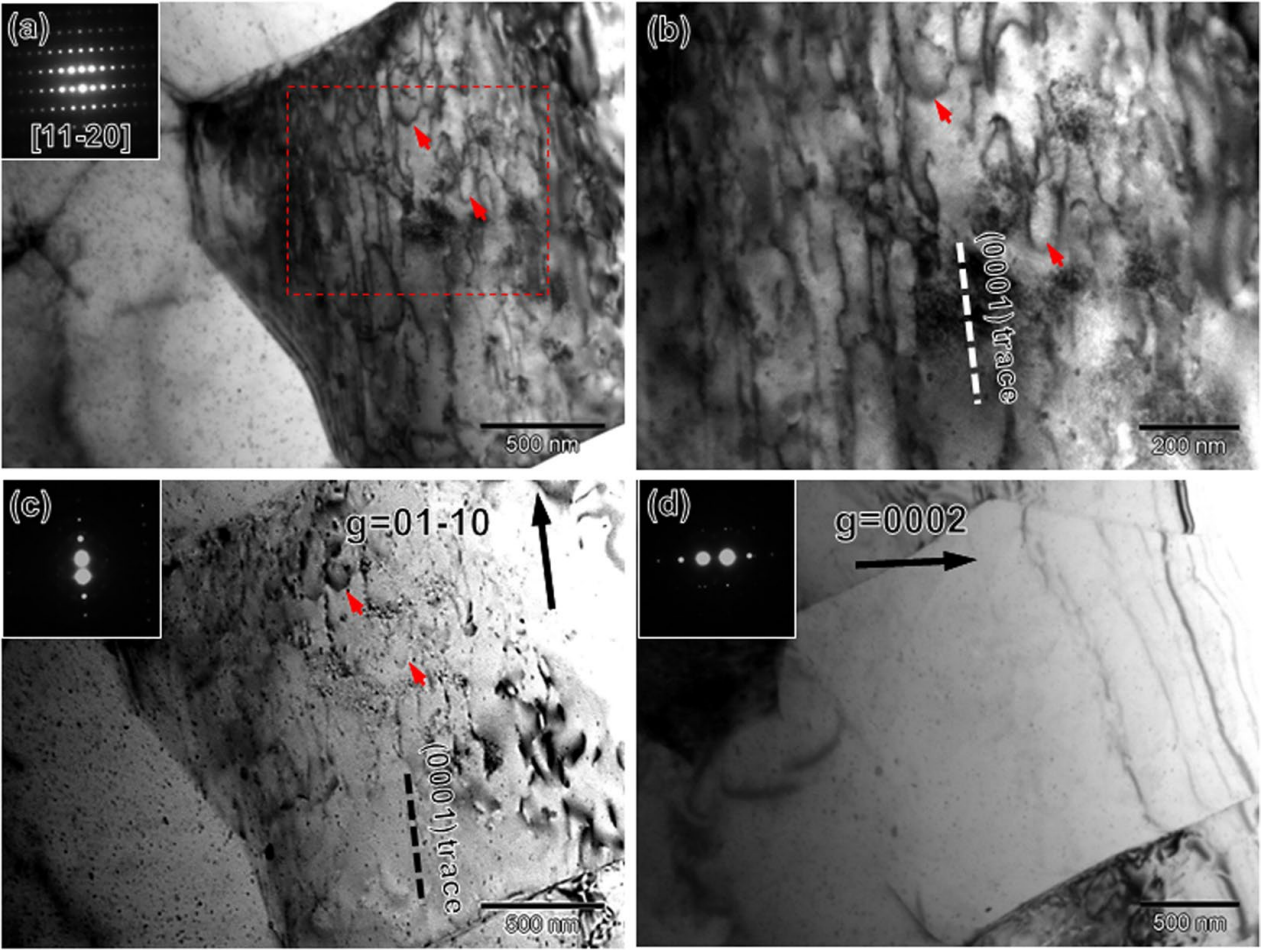
Dislocation structures of a typical grain in the UFG specimen (*d* = 0.98 μm) tensile deformed to a strain of 0.02. (**a**) BF-TEM image observed along^11–20^ zone axis, showing profuse dislocation structures. (**b**) BF-TEM image of the red broken rectangular area in (**a**) at higher magnification. (**c**) BF-TEM image observed under two beam condition with ***g*** = 01–10. (**d**) BF-TEM image observed under two beam condition with ***g*** = 0002.

Since it was quite difficult to recognize slip lines (like Fig. 6(c)) in ultrafine grains even at small strains, we conducted TEM observations of the UFG specimen tensile deformed to several specified strains. Figure 9 represents a grain in the UFG specimen after 0.02 tensile deformation, observed from near the^11–20^ zone axis by a minimum tilting. As indicated by the SAED pattern inserted in Fig. 9(a), the (0001) basal plane was aligned parallel to the incident beam direction^38^. Figure 9(b) is an enlarged view of the boxed region in **(a**), from which profuse dislocations aligned roughly parallel to the basal trace (the broken line in **(b)**) were observed. These dislocations were most likely basal dislocations. Besides, some curved dislocation segments (indicated by red arrows) that were not parallel to the basal trace were also observed, indicating some non-basal slip systems were also activated. In order to know the Burgers vector of these dislocations, this grain was further observed from near the^11–20^ zone axis under two beam conditions. The operation vector (***g***) in Fig. 9(c,d) were 01–10 and 0002, respectively. As shown in Fig. 9(c), dislocation configurations were still observed, and they showed a good correspondence with the dislocation structures observed in Fig. 9(a). In contrast, Fig. 9(d) was did not show any dislocation contrast, suggesting that all the dislocations in Fig. 9(a) have Burgers vector parallel to <***a***>. We also checked many other grains and they all exhibited similar dislocation configurations. Thus, we can conclude that the non-basal slip system having <***c***> component such as {11–22} second-order pyramidal slip was not activated at this strain level. The dislocation segments that were not parallel to the basal trace (Fig. 9(b)) are considered to be <***a***> type dislocations cross-slipped from the basal plane to other slip planes, such as {10-10} prismatic plane.

**Figure 10:**
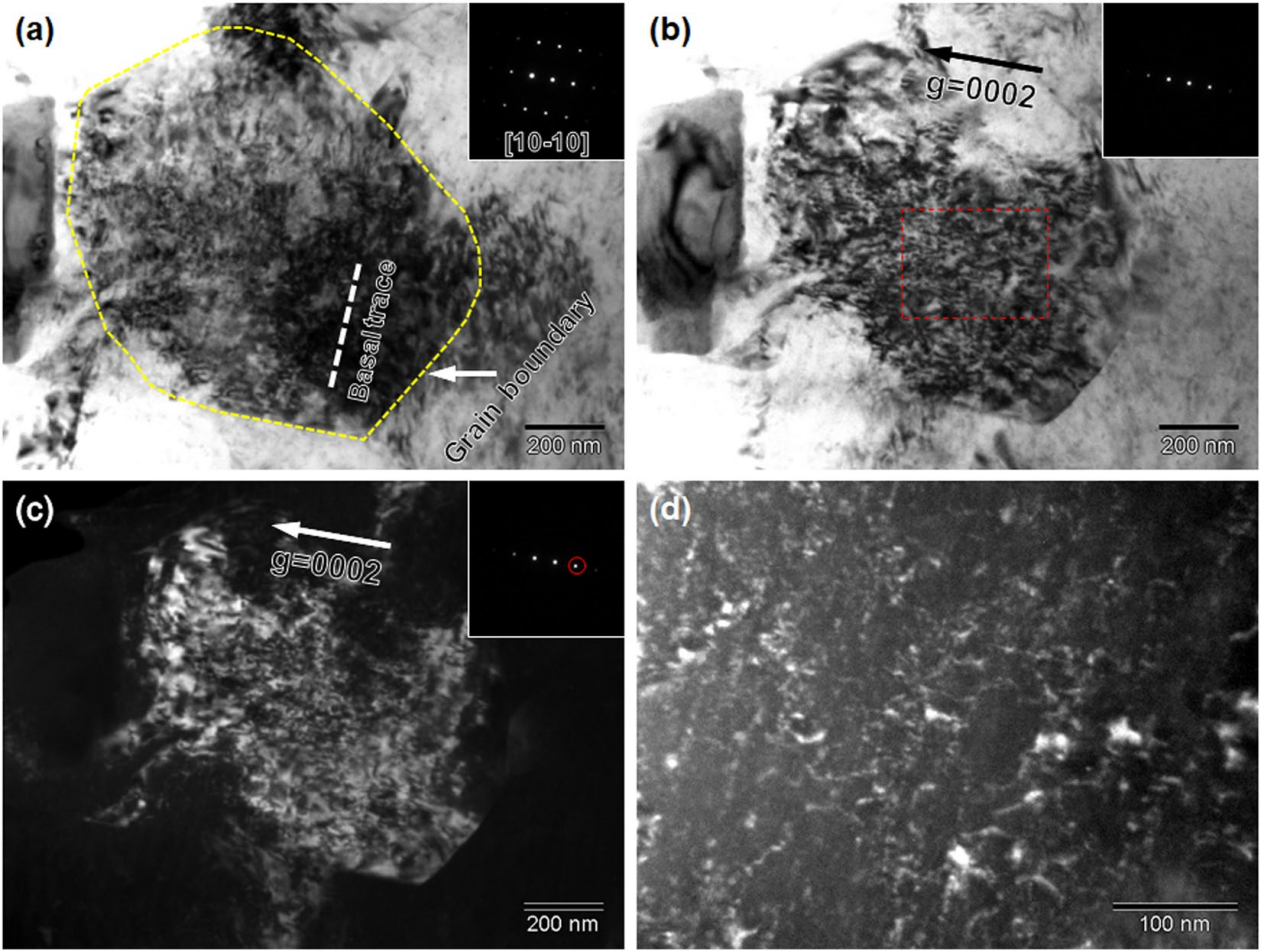
Dislocation structures of a typical grain in the UFG specimen (*d* = 0.98 μm) tensile deformed to a strain of 0.095. (**a**) BF-TEM image observed along [10-10] zone axis. (**b**) BF and (**c**) corresponding DF-TEM images observed under two beam condition with ***g*** = 0002. (**d**) WBDF image of the rectangular area in (**b**) at higher magnification.

The UFG specimen was further observed at a tensile strain of 0.095 by TEM. Figure 10 shows a grain observed from near the [10-10] zone axis (see inserted SAED pattern) by minimum tilting, where the grain boundary was marked by yellow dashed line. Under this beam condition, the basal plane was also edge-on, so it was again possible to distinguish the types of dislocations based on the “***g*** · ***b*** **=** 0” criterion. The dislocations in Fig. 10(a) were not straight like Fig. 9(a) but tangled very much. Through additional minimal tilting, this grain satisfied two beam condition. A BF- TEM image and corresponding dark field (DF) TEM image under two beam condition with ***g*** **=** 0002 (see inserted SAED patterns) were shown in Fig. 10(b,c), respectively. Interestingly, a high density of dislocations were still observed, suggesting that these dislocations had <***c***> component. These dislocations were further observed at higher magnification under a weak-beam dark field (WBDF) condition (Fig. 10(d)) through a so-called ***g***(3 ***g***) operation^40^. The diffraction contrast (dislocation segments having bright contrast) in Fig. 10(d) confirmed that dislocations having <***c***> component were activated at this strain level in the UFG specimen. Therefore, we can conclude that deformation twinning was significantly suppressed throughout the entire deformation process in the UFG specimens, whereas the non-basal slip systems having <***c***> component Burgers vectors were activated to realize three-dimensional plastic deformation. The activation of the unusual slip systems is considered to contribute to the enhanced work-hardening rate in the UFG specimen, as is discussed in the next section.

## Discussion

**Figure 11:**
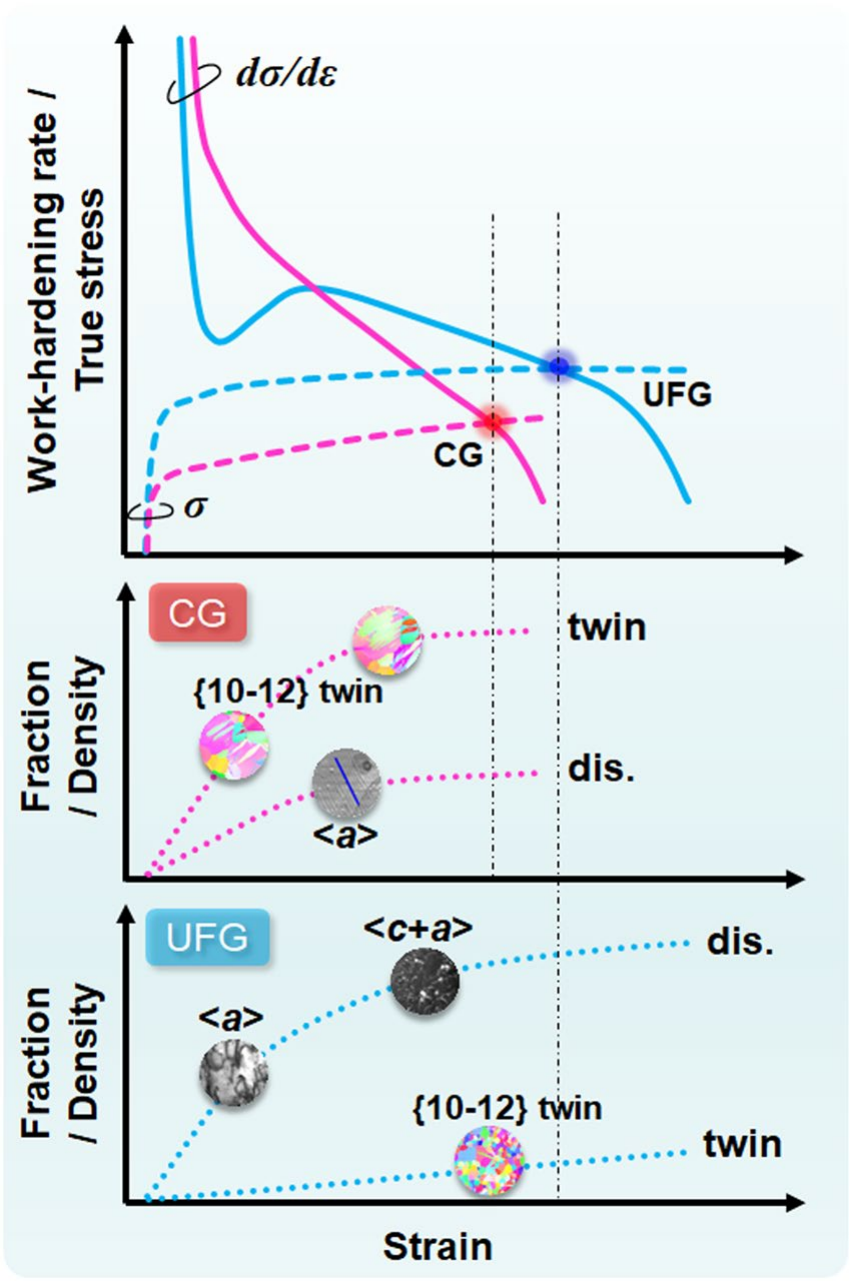
Schematic illustration showing the correlation between the work-hardening behavior and the evolution of dominated deformation modes (deformation twinning and dislocation slip) at different strain levels in CG and UFG specimens.

Based on the systematic observations of deformation microstructures (Figs 5–10**)**, the relationship between the work-hardening behavior and the dominated deformation modes at various strain levels in the CG and UFG specimens is schematically revealed in Fig. 11. In the CG specimen, the plastic deformation was predominated by {0001} <11–20> basal slip and {10–12} <10–11> twinning. The relatively high work-hardening rate at the early stage is due to the rapid growth of {10–12} <10–11> twinning, which can be understood in the following ways: (i) twinning introduces additional barriers (namely, twin boundaries) for subsequent dislocation slips, thus it is somehow equivalent to dynamic refinement of the grain size; (ii) {10–12} <10–11> twinning turns the lattice to a new orientation which is “hard” for subsequent plastic deformation^41–45^. Afterward, the subsequent quick decrease of work-hardening rate is due to the deceleration and saturation of deformation twinning at the high strain level, as confirmed in Fig. 5. In contrast, deformation twinning was dramatically suppressed in the UFG specimen. At the very beginning of plastic deformation, the V-shaped valley in the work-hardening rate curve is associated with the discontinuous yielding phenomena including yield-drop and subsequent Lüders deformation in fully recrystallized UFG specimen. Basal and non-basal slip systems having only <***a***> component dominated the plastic deformation at the early stage. Subsequently, <***c*** + ***a***> dislocations were activated, which played a crucial role in strengthening the material, especially in producing deformation along the <***c***> axis. Additionally, activation of dislocations having totally different Burgers vectors would enhance interactions between dislocations, leading to an increase of work-hardening^46^, which was supported by the high density of tangled dislocations observed in Fig. 10. The enhanced work-hardening results in not only high strength but also large elongation by postponing the plastic instability criterion.

It should also be noted that, in addition to the activation of unusual slip systems, some other mechanisms such as grain boundary sliding are also expected to be important in UFG Mg, which could improve the ductility or formability of Mg. For example, Zeng *et al*.^47^ reported that a bulk specimen of pure Mg with a mean grain size of about 1 μm could be compressed to a large strain of 85% without fracture. Their observation of deformation microstructures and strain rate jump test suggested that intergranular deformation modes such as grain boundary sliding were responsible for the good formability of the UFG pure Mg. However, for the highly alloyed Mg-Zn-Zr-Ca alloy studied in the present study, the situation is greatly different. As shown in Fig. 1, we found that the alloying elements (especially Zn) strongly segregated to the newly formed grain boundaries of ultrafine grains, which could effectively stabilize grain boundaries and reduce their mobility. It is difficult to imagine that such grain boundaries decorated by segregated elements show grain boundary sliding. Moreover, the UFG ZKX600 alloy specimens showed an excellent work-hardening behavior, which also denies a possibility of grain boundary sliding, since grain boundary sliding basically results in flow softening rather than hardening.

It is an important and new finding that unusual slip systems having <***c***> component could be activated when the grain size was refined down to sub-micrometer scale. Consequently, synergetic improvement of strength and ductility was realized in the UFG specimen. The reason for the activation of unusual slip systems is still unclear. It is noteworthy, however, that UFG materials that can manage both high strength and large ductility have been recently found in particular alloys such as Cu-Al^48,49^ and high-Mn steels^50,51^, although it was considered that ultra grain refinement generally leads to the decrease of uniform elongation owing to early plastic instability. For example, in Cu-Al alloys having single-phased FCC structure with very low stacking fault energies, deformation twinning was found to be enhanced in fully recrystallized UFG microstructures^49^, which was totally opposite to the well-known grain size dependence of deformation twinning, *i.e*., deformation twinning becomes difficult to occur with decreasing the grain size. All those results suggest that there is an unknown mechanism to enhance unusual deformation modes in fully recrystallized UFG structures.

## Conclusions

In summary, the change of mechanical properties and deformation mechanisms in fully recrystallized UFG specimens were systematically investigated. We found that: (i) the plastic deformation of the coarse grained specimen (*d* = 23.3 μm) was predominated by {10–12} <10–11> twinning and {0001} <11–20> basal slip, and the quick decrease of work-hardening rate at the later stage of plastic deformation was due to the deceleration and saturation of deformation twinning, (ii) deformation twinning was dramatically suppressed in the UFG specimen (*d* = 0.98 μm), while non-basal slip systems having <***c***> component Burgers vectors were activated successively. Particularly, the operation of the <***c***> component dislocations was responsible for the enhanced work-hardening rate. The present results showed a new insight that hexagonal Mg alloys can be very ductile by activating unusual deformation mechanisms (<***c*** + ***a***> dislocations) through realizing fully recrystallized UFG microstructures.

## Methods

A Mg-6.2%Zn-0.5%Zr-0.2%Ca (mass%) alloy produced by induction melting in Ar was used as the starting material. The as-cast alloy was provided for a multi-step thermo-mechanical processing, including solution treatment (ST), HPT to various rotation angles, and subsequent annealing treatment. The details of processing can be found in our previous publication^17^. The principle of the HPT process and coordinate in the HPT processed disc are schematically shown in Fig. S2(a,b), respectively.

Microstructures of the specimens having various grain sizes were examined using scanning electron microscopy (SEM) and electron backscattering diffraction (EBSD). The specimens for SEM and EBSD analysis were mechanically polished using a fine grade sandpaper followed by a final polish using a colloidal silica suspension (OP-S, Struers). EBSD results were analysed by TSL OIM software. Dislocation structures were observed by transmission electron microscopy (TEM). TEM specimens were firstly mechanical polished to 100 μm in thickness and then twin-jet electropolished using a solution of 5.3 g LiCl, 11.6 g Mg(ClO_4_)_2_, 500 ml methanol and 100 ml 2-butoxy-ethanol at a voltage of 65 V and temperature approximately −50 °C.

Mechanical properties of the specimens having various grain sizes were characterized by tensile test with an initial strain rate of 8.3 × 10^−4^ s^−1^. The selected positions for microstructure observations and tensile tests as well as the detailed dimensions of the tensile specimen are shown in Fig. S2(c). Digital image correlation (DIC) technique was applied to precisely measure the tensile strain. The DIC technique was also used to observe local strain distribution within the gage length of the specimens during the tensile test. For each processing condition, three tensile specimens were tensile tested for a particular microstructure, in order to ensure the reproducibility of obtained stress-strain curves.

## Supplementary information

Supplementary Information
