# Supplementary material for: Change of Deformation Mechanisms Leading to High Strength and Large Ductility in Mg-Zn-Zr-Ca Alloy with Fully Recrystallized Ultrafine Grained Microstructures
Source: Sci Rep. 2019 Aug 12;9:11702. doi: 10.1038/s41598-019-48271-5 (PMC6691010; doi:10.1038/s41598-019-48271-5)
Supplement: Supplementary file 1 — Supplementary Information [file 41598_2019_48271_MOESM1_ESM.docx]

***Supplementary information for***

**Change of Deformation Mechanisms Leading to High Strength and Large Ductility in Mg-Zn-Zr-Ca Alloy with Fully Recrystallized Ultrafine Grained Microstructures**

Ruixiao Zheng^1,2,*^, Tilak Bhattacharjee^2,3^, Si Gao^2^, Wu Gong^3^, Akinobu Shibata^2,3^, Taisuke Sasaki^4^, Kazuhiro Hono^4^, Nobuhiro Tsuji^2,3,*^

^1^ School of Materials Science and Engineering, Beihang University, Beijing 100191, China

^2^ Department of Materials Science and Engineering, Kyoto University, Yoshida Honmachi, Sakyo-ku, Kyoto 606-8501, Japan

^3^ Elements Strategy Initiative for Structural Materials (ESISM), Kyoto University, Yoshida Honmachi, Sakyo-ku, Kyoto 606-8501, Japan

^4^ National Institute for Materials Science, 1-2-1 Sengen, Tsukuba 305-0047, Japan

*Corresponding author: (R. X. Zheng) zhengruixiao@buaa.edu.cn

(N. Tsuji) nobuhiro-tsuji@mtl.kyoto-u.ac.jp


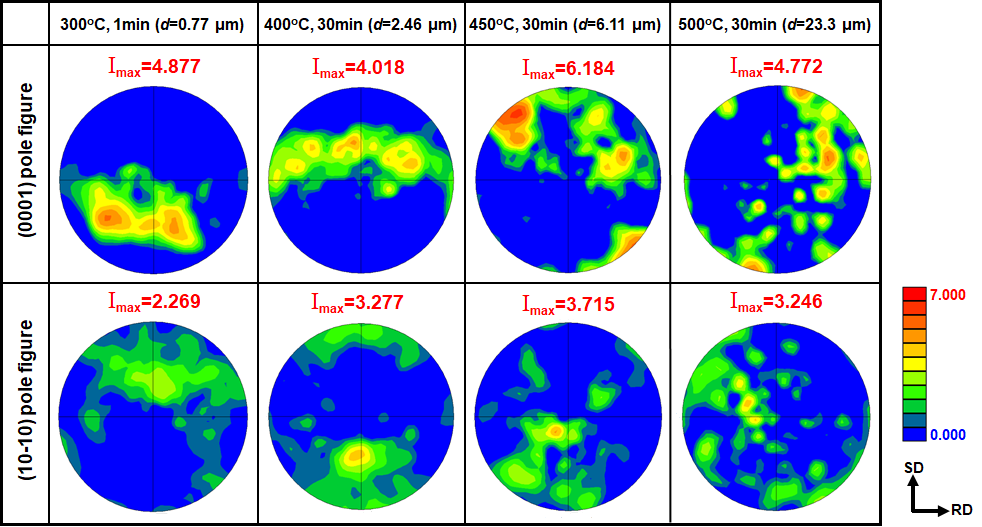


**Figure S1** (0001) and (10-10) pole figures of the fully recrystallized specimens with various mean grain sizes (*d*).

**
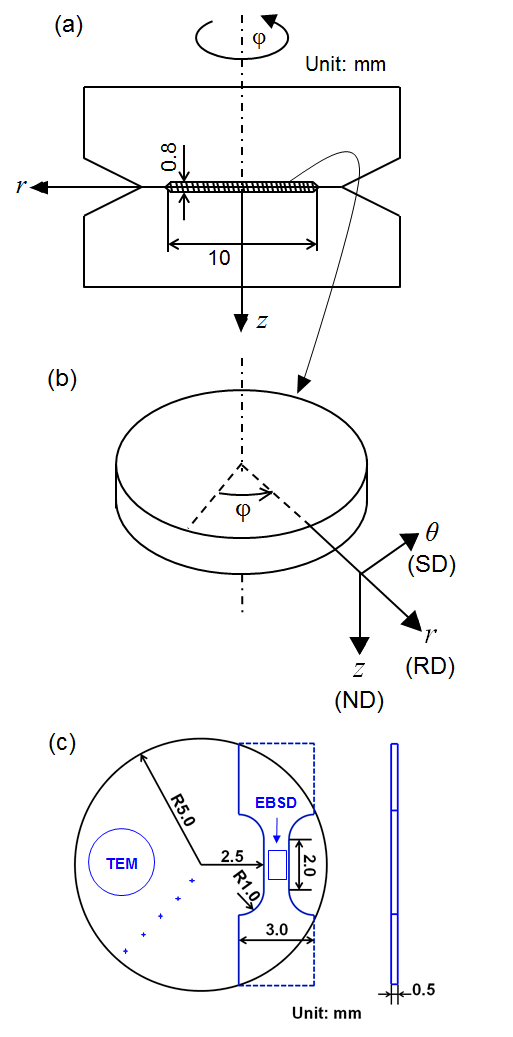
**

**Figure S2** Schematic illustrations showing (a) the principle of the HPT process, (b) the coordinate in the HPT processed disc and (c) the selected positions for microstructure observations and tensile tests. SD, RD and ND in (b) represent the shear direction, radial direction and normal direction, respectively.
